# Patients’ knowledge about medicines improves when provided with written compared to verbal information in their native language

**DOI:** 10.1371/journal.pone.0274901

**Published:** 2022-10-31

**Authors:** Rameshkumar T., Haputhanthrige I. U., Misbahunnisa M. Y., Galappatthy P.

**Affiliations:** 1 Department of Pharmacology, Faculty of Medicine, University of Colombo, Colombo, Sri Lanka; 2 School of Pharmacy, National Hospital of Sri Lanka, Colombo, Sri Lanka; PLOS (Public Library of Science), UNITED KINGDOM

## Abstract

**Background:**

Patient’s knowledge on the medicines prescribed is vital in reducing medication errors and improving adherence and patient satisfaction. This study assessed the patients’ knowledge on medicines prescribed and compared improvement following verbal or written information given in patients own language.

**Methods:**

Seventy-five trainee pharmacists randomised to verbal or written groups, provided information to consecutive consenting patients attending medical and surgical clinics in the outpatient department of National Hospital of Sri Lanka. Baseline knowledge and immediate recall knowledge was scored on 5 categories (name, indication, dose, frequency of administration, and additional relevant details) for each drug prescribed. Scores <5/10, 5-7/10 and > 7/10 were considered as poor, moderate, and good knowledge. Sample of 384 in each group had 80% power to detect a 10% difference in knowledge level. Data was analysed using SPSS v26.

**Results:**

Of the 747 patients, 429(57%) were females, mean age was 54.3 years(SD±12), 450(60%) were on 5 or more drugs and 149(20%) were elderly (>65 years). At baseline, knowledge scores were comparable in both groups with 47% (352/747) patients having poor knowledge, 34% (254/747) moderate knowledge and only 19% (141/747) good knowledge. Patients knew the name only on 1653(42%) drugs prescribed, indication on 1603(41%), dose on 860(22%), administration details on 2255(58%) and additional details (adverse effects/storage) only on 267(7%) medicines prescribed. After the intervention, 276(74%) patients had good knowledge (score>7), in written group vs 217(58%) in verbal group (p<0.05). In all 5 aspects knowledge improved significantly more in written group compared to the verbal group. Binomial logistic regression analysis indicated that ordinary level (OR:2.32), advanced level(OR:2.13) and university education(OR:3.72), and lower number of medicines prescribed(OR:0.83) significantly increased the odds of having a “Good Knowledge on Medicines”.

**Conclusion:**

Patient’s knowledge on medicines prescribed was poor and providing the medication plan in writing in patients’ own language would improve the knowledge significantly compared to providing verbal information.

## Introduction

Medicines are a key component of patient care and providing clear instructions on medicines to improve knowledge among patients is important to reduce medication errors. If medicines are not accurately taken by patients, significant morbidity and even mortality can occur. It is estimated that at least 1.5 million preventable medicine-related adverse events occur in the USA per year, with medicine prescribing, administering, and dispensing errors contributing to the majority [1]. The Australian Commission on Safety and Quality in Health Care reports that 2–3% of all hospital admissions are medication related and this rises to 20–30% in the population aged >65 years [2]. Globally, medication errors are estimated to cost US$ 42 billion annually [3]. World Health Organization launched ‘Medication Without Harm’ as its third Global Patient Safety Challenge. The challenge is to reduce the level of severe, avoidable harm related to medications by 50% over five years globally [4, 5]. Improving the knowledge of patients and the public on medicines is one of the 4 subdomains identified in the challenge [6].

The patient is the last link in the medication administration chain and the final individual capable of preventing incorrect medication usage [7]. An important aspect of prescribing and dispensing medicines is adequately educating the patients on the medicines they are given. Providing information on medicines to patients can improve adherence and patient satisfaction, prevent the incidence of therapeutic duplication and drug interactions, and prevent potentially fatal adverse drug reactions [8]. Patient knowledge on medications has been defined as awareness of the drug name, purpose, administration schedule, adverse effects or special instructions [9]. Studies have shown that low health literacy results in poor health outcomes, particularly in misunderstanding of instructions on medication use [10, 11].

Adequate knowledge of patients on the medicines they are taking would improve safety. Although there are novel ways of educating patients on medicines prescribed using methods such as mobile applications and pictograms, common methods that are universally applied are either providing verbal instructions or written information, particularly in low and middle-income countries (LMICs).

In such resource-limited settings, patient’s level of knowledge on medicines they take is often poor and language barriers in communication also contribute to poor knowledge and medication errors. In most countries, particularly the commonwealth, the healthcare professionals are trained in English, and labelling of medicines and writing discharge summaries is done in English, which most patients may not understand. In such settings, with overcrowding of patients, there is very limited time to provide information, even on the basic instructions on medicine use. In a previous study conducted in Sri Lanka, limited English proficiency, lower level of education and lack of perception of illness severity were shown to reduce the knowledge of prescribed medications [12].

Therefore, this study aimed to compare the change in patients’ knowledge about the medications prescribed, with verbal information vs written information provided in patients own language, by trainee pharmacy students at outpatient clinics.

## Methods

We conducted a cross sectional study among patients at outdoor pharmacy of National Hospital of Sri Lanka (NHSL) during the months of September and October 2016. Patients who could not provide consent, understand or express their views because of a speech/language disability or a hearing defect or psychiatric disorder were excluded from the study. The study was approved by the Ethics review committee of the Faculty of Medicine, University of Colombo. Written informed consent was obtained before the interview.

A sample size calculated using the software (available in http://hedwig.mgh.harvard.edu/sample_size/size.html), was 384 per group to determine a significant difference in the two means of 1 point with a standard deviation of 5 points at 80% power with a two-sided p-value of less than 0.05. Data collection was done by 75 trainee pharmacists in their final year of training attached to School of Pharmacy, NHSL. Trainee pharmacists were randomly allocated by drawing lots among them to give either written or verbal information to patients. We included 38 scrolls indicating ‘verbal’ and 38 indicating ‘written’, given in a basket and each trainee pharmacist was asked to pick one scroll and was allocated to the group indicated in the scroll. Each trainee pharmacist recruited consecutive 10 patients to the study after obtaining informed written consent. Interviewer administered questionnaire was used for data collection. Baseline knowledge on medicines was assessed under 5 areas for each medicine separately and average knowledge score was calculated, and the scoring was done as indicated in S1 Table. Knowledge score for each patient was calculated by taking the average of the scores of individual medicines taken. (e.g. If a patient was on n number of medicines, the total knowledge score for the patient was divided by n to get the average score for each patient)

Poor, moderate, and good levels of knowledge was considered when the average knowledge score was <5, 5–7, >7, respectively.

Information on medicines was provided by the interviewing trainee pharmacist either in Sinhala or Tamil, the two languages used mostly by patients. Any student who encountered a Tamil/Sinhalese patient got the help of a Tamil/Sinhalese speaking student to provide information if he or she was not conversant in written/verbal language of the patient. Following the initial interview, the same interviewer educated the patient on above five areas for each drug in writing or verbally. The same interviewer using the same scoring system checked immediate recall knowledge on the medicines they were prescribed.

SPSS (Statistical Package for Social Sciences) version 26 was used in data analysis. Proportions and means were used to describe categorical and continuous variables respectively. Binary logistic regression was used to assess the factors associated with “Good Knowledge on medicines”. Chi square tests were used to compare the significance of knowledge before and after the intervention and the effect of verbal vs written information in improving patient’s immediate recall and understanding. Student t test was used for comparison of continuous variables. Statistical significance was determined at the p value <0.05.

## Results

**Fig 1:**
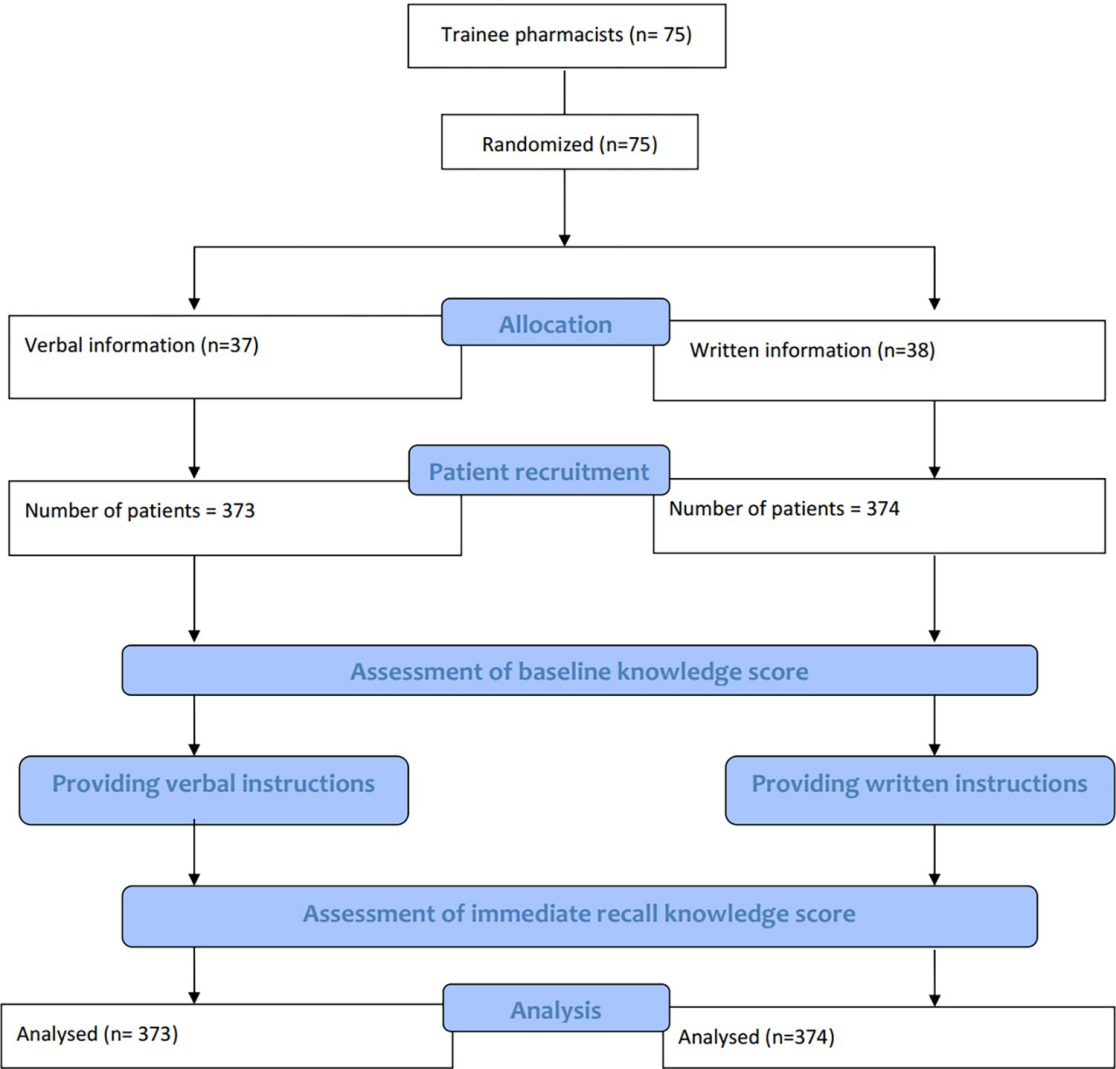
Flow diagram on number of trainee pharmacists and patients allocated to the two groups following randomization.

Of the total of 747 patients recruited (Fig 1), 429 (57%) were females with a mean age of 54.3 years and 149 (20%) were categorised as elderly, over 65 years. Their level of education, number of medicines prescribed and baseline knowledge on each aspect of the medicines are given in Table 1.

**Table 1:** Baseline characteristics of the study subjects (N = 747).

Variable	Total sample (N = 747)	Verbal group (n = 373)	Written group (n = 374)	P value
Age, years	54.3 ±12	54.2 ± 12.5	54.5 ±12	0.724^±^
Male	318 (43%)	154 (41%)	164 (44%)	0.479*
Female	429 (57%)	219 (59%)	210 (56%)	
Education				
Below Grade 5	94 (13%)	48 (13%)	46 (12%)	0.891*
Between Grade 5 and GCE (O/L)s	193 (26%)	97 (26%)	96 (26%)	
Between GCE (O/L)s and (A/L)s	270 (36%)	136 (36%)	134 (36%)	
Passed GCE (A/L)s	156 (21%)	78 (21%)	78 (21%)	
University Education	34 (5%)	14 (4%)	20 (5%)	
Number of medicines prescribed				
1	6 (1%)	5 (1%)	1 (0.3%)	0.007^≠^
2	34 (5%)	15 (4%)	19 (5%)	
3	76 (10%)	46 (12%)	30 (8%)	
4	181 (24%)	103 (28%)	78 (21%)	
5 or more	450 (60%)	204 (55%)	246 (66%)	
9 or more	35 (5%)	12 (3%)	23 (6%)	
Mean number of medicines	5.2 ± 1.8	5 ± 1.8	5.5 ± 1.9	<0.001^±^
Knowledge on medicines	5.1 (±1.9)	5 (±1.9)	5.2 (±2)	0.083^±^
Mean score on name	1.2 (±0.5)	1.2 (±0.5)	1.1 (±0.5)	0.681^±^
Mean score indication	1 (±0.6)	1 (±0.6)	1.1 (±0.6)	0.012^±^
Mean score on dose	1 (±0.5)	1 (±0.5)	1 (±0.5)	0.405^±^
Mean score on frequency	1.4 (±0.6)	1.4 (±0.6)	1.5 (±0.6)	0.023^±^
Mean score on additional information	0.4 (±0.5)	0.3 (±0.4)	0.4 (±0.5)	0.009^±^
Number of patients with good knowledge on each aspect**				
Name (Percentage)	96 (13%)	51 (14%)	45 (12%)	0.503*
Indication (Percentage)	84 (11%)	39 (11%)	45 (12%)	0.495*
Dose (Percentage)	62 (8%)	34 (9%)	28 (8%)	0.420*
Details of administration (Percentage)	308 (41%)	143 (38%)	165 (44%)	0.109*
Additional details (Percentage)	12 (2%)	4 (1%)	8 (2%)	0.384^≠^
Overall Knowledge Category				
Poor	352 (47%)	188 (50%)	164 (44%)	0.183*
Moderate	254 (34%)	121 (32%)	133 (36%)	
Good	141 (19%)	64 (17%)	77 (21%)	

Statistical tests used are Chi-square (*), Fisher’s Exact test (^≠^) and student t-test (±)

**Patients who score 2 on individual aspects on all medicines prescribed

Total of 3901 medicines were prescribed for the patients and the baseline knowledge level was generally poor, with mean total score 5.1 and 47% having poor level of knowledge. Scoring for knowledge areas assessed for individual drugs is given in Table 2.

**Table 2:** Baseline scores obtained for each knowledge component for each medicine prescribed (N = 3901).

Knowledge Area	Score		
	0	1	2
Name of the drug	1095 (28%)	1153 (30%)	1653 (42%)
Indication for the drug	1383 (35.5%)	915 (23.5%)	1603 (41%)
Dose of the drug	742 (19%)	2299 (59%)	860 (22%)
Details of administration	573 (15%)	1073 (27%)	2255 (58%)
Additional details	2795 (72%)	839 (21%)	267 (7%)

A binomial logistic regression was performed to ascertain the effects of age, gender, education level and the number of medicines prescribed on the likelihood of patients’ having good knowledge on the medicines. The logistic regression model was statistically significant, χ2 = 35.031, p < .0005. The Cox & Snell R-Square and Nagelkerke R Square values were 0.046 and 0.074, respectively. Of the four predictor variables two were statistically significant: level of education and number of medicines prescribed as shown in Table 3. Results indicate that having passed O/Ls (OR: 2.32, 95% CI 1.15–4.65), having passed A/Ls (OR: 2.13, 95% CI 1.01–4.5), university education (OR: 3.72, 95% CI 1.41–9.79) and lower number of medicines prescribed (OR: 0.83, 95% CI 0.75–0.94) significantly increased the odds of having a “Good Knowledge on Medicines”.

**Table 3:** Binary logistic regression analyses of factors predicting “Good Knowledge on medicines”.

	Odds ratio	95% Confidence Interval	P value
Age	0.99	0.98–1	0.336
Female gender (0 = Male)	0.92	0.63–1.34	0.656
Level of Education (0 = Below Grade 5)			
Above grade 5 and below O/L	0.94	0.43–2.04	0.866
Passed O/Ls	2.32	1.15–4.65	0.018
Passed A/Ls	2.13	1–4.5	0.048
University education	3.72	1.41–9.79	0.008
Number of Medicines	0.83	0.75–0.94	0.002

**Fig 2:**
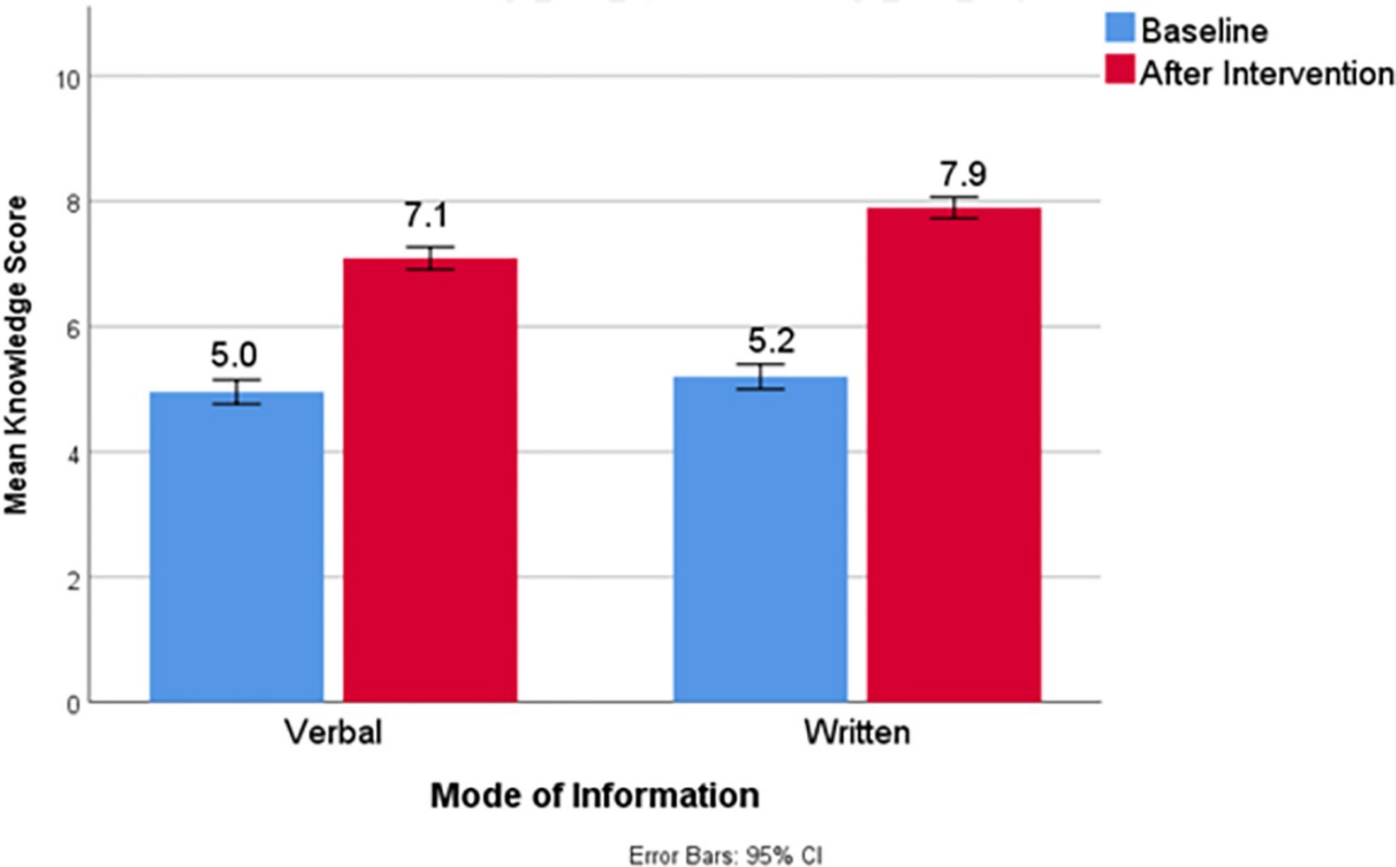
Comparison of mean knowledge score between written and verbal instruction groups.

At baseline, both verbal and written groups had similar levels of knowledge but after instructions, the group which was given written instructions had significantly lower number of poor scorers (Table 4). The improvement in mean knowledge score was significant in both groups after providing instruction (Fig 2). The knowledge assessed for each drug improved significantly in all 5 aspects, after written instruction compared to verbal instructions (Table 5).

**Table 4:** Comparison of knowledge score between written and verbal instruction group.

Type of intervention	Verbal Instruction		Written Instruction		P value
Knowledge category	Baseline	After intervention	Baseline	After intervention	
Poor	188 (50%)	49 (13%)	164 (44%)	28 (8%)	<0.001*
Moderate	121 (32%)	107 (29%)	133 (36%)	70 (19%)	
Good	64 (17%)	217 (58%)	77 (21%)	276 (74%)	

Statistical test used is Chi square (*).

**Table 5:** Comparison of knowledge components after written and verbal instruction in the two groups.

Score obtained under each knowledge area	Number (%) of medicines with knowledge at Baseline	Number (%) of medicines with knowledge after verbal instruction	Number (%) of medicines with knowledge after written instruction	P value
Name of the drug				<0.001*
0	1095 (28%)	207 (11%)	135 (7%)	
1	1153 (30%)	421 (23%)	399 (20%)	
2	1653 (42%)	1229 (66%)	1510 (74%)	
Indication for the drug				<0.001*
0	1383 (36%)	146 (8%)	76 (4%)	
1	915 (23%)	538 (29%)	426 (21%)	
2	1603 (41%)	1173 (63%)	1542 (75%)	
Dose of the drug				<0.001*
0	742 (19%)	107 (6%)	92 (4%)	
1	2299 (59%)	781 (42%)	502 (25%)	
2	860 (22%)	969 (52%)	1450 (71%)	
Administration details				<0.001*
0	573 (15%)	65 (4%)	64 (3%)	
1	1073 (27%)	492 (26%)	315 (15%)	
2	2255 (58%)	1300 (70%)	1665 (82%)	
Additional details				<0.001*
0	2795 (72%)	839 (45%)	577 (28%)	
1	839 (21%)	661 (36%)	880 (43%)	
2	267 (7%)	357 (19%)	587 (29%)	

Statistical test used is Chi squarer (*).

## Discussion

This study was done to identify change in knowledge on medicines prescribed to patients, provided verbally in their native language in comparison to written language. We found that the baseline minimum knowledge of patients should have on medicines prescribed is low with only 19% having a good level of knowledge (categorised as a value above 7) with a mean value of 5.24 out of 10 points. The knowledge significantly improved with both verbal and written information while the improvement was significantly higher with written information compared to verbal information.

The patients were more knowledgeable on frequency of medicine administration and the name of the medicine and less knowledgeable on the exact dose of the drug and additional details like adverse effects at baseline. A study by Frank J. et al, also showed similar results with participants being more knowledgeable on administration schedule and dose but having less knowledge on indication of the medicine and adverse effects [9]. In a study done in Bangladesh, only 12% of participants identified the medicines by their generic names but the dosage regimen and the administration details was known by 90% of participants [13].

High level of knowledge on frequency of administration is expected as patients would know how many times, they take their regular medicines. Participants’ poor knowledge on additional details such as adverse effects and storage would be due to the less time available for physicians and pharmacists to provide that information while essential information such as information on drug administration are provided.

Regarding drug dose, majority (59%) knew the number of pills taken than the actual dose in grams/ milligrams. This can lead to difficulties in obtaining drug history in clinic visits and when different strengths of the same drug are available which could also lead to drug overdosing or under dosing.

In our study, the factors which had a positive effect on the knowledge level were less number of medicines prescribed and higher level of education. Ahmet Akici and et al, in Turkey found that higher level of education, female gender, having chronic disease had a positive impact on recalling drug names [14].

Only 42% of the medicines prescribed were correctly named by the participants. In the study by Ahmet Akici and et al. in Turkey in a primary care setting, only 9.5% of the medicines were correctly named [14]. Vilke G.M. and et.al. in their study in Emergency Department in US, 48% of the patients could recall the names of the medicines [15]. Jaye C. and et al. reported that in a General Practice setting in New Zealand, 85% of the medicine names were correct [16]. A study in Spain in 2011 revealed that that only 19.2% (45/234) of the medicines prescribed to inpatients were known and in the outpatient group, 69% (29/42) of the medicines were known [17]. The difference in this could be accounted by difference in care setting, the native language of the country and by the educational level of the participants.

In Sri Lanka doctors and pharmacists study medicine in English and labelling of medicines dispensed to patients is also done in English while most patients particularly attending government hospitals speak the native languages, Sinhala and Tamil. This does not help to improve patients’ knowledge of the medicines as most of the patients attending particularly public sector hospitals do not have adequate knowledge of English. Studies conducted in Sri Lanka have shown that medium of language in written information influences patients’ knowledge [12, 18]. Studies by Perera et al. showed that majority of the patients were unable to read and understand the information written in English [12] and 92% patients prefer the discharge summaries given in native language for better understanding [18]. Importance of native language in health communications have been shown in other settings as well [19].

This study showed that even simple measures such as providing verbal or written instructions in native language are effective in adequately empowering the patient to identify their medicines with regards to basic details like the name of the drug, the dose, frequency, and specific instructions while using them. Taking steps to provide drug labels in native languages, providing the information on medicines (drug name, indication, dose, frequency, and timing) using printed labels or seals can be done even in resource limited settings with committed staff even with existing resources. Establishing these practices especially in LMICs can contribute to improvement of patients’ knowledge on medicines. One key focus area to reduce medication errors recommended by WHO in the third global patient safety challenge on medication without harm is improving patients’ knowledge of medicines. Providing written information in native language in medicines labels is one action that can be implemented without many resources even in resource limited settings.

Present study has several limitations. We have not collected some information such as whether patient was given information about the drugs by a health care worker or the length of time the patient has been taking each drug which could affect their knowledge. These could affect the baseline level of knowledge of patients reported. Also, the study was done in a tertiary care teaching hospital and would not represent the knowledge of patients who are being treated at primary care centres. The knowledge was assessed by immediate recall after providing the information and whether that would be retained longer term particularly with verbal information is doubtful. Also, the information was provided by trainee pharmacists with clear labels and patients interest and understanding might be even greater if given by dispensing pharmacist. The details of the underlying disease for which medicines were taken was not collected in the study which could also affect the patients’ knowledge on medicines. We have excluded patients with speech/ language difficulties, hearing disabilities and psychiatric patients who form an important group with many difficulties in communication and are vulnerable to medication errors. Further studies are needed to compare the knowledge of patients and the interventions to improve it across different levels of health care delivery and those with communication and other different disabilities.

## Conclusion

This study demonstrated that knowledge of patients regarding their medicines was low and was positively influenced by the level of education, and lower number of medicines prescribed. Both verbal and written information provided in patients own language on medicines improved knowledge, but written information improved knowledge significantly more compared to verbal information and in comparison, to the baseline. Medication plan (name, dose, frequency and indication of each medicine prescribed) should be given preferably in writing to patients in their own language, to improve patient knowledge on medicines.

## Supporting information

S1 Table Scoring system used to assess the knowledge on the medicines prescribed. (PDF)
