# Supplementary material for: Patients’ knowledge about medicines improves when provided with written compared to verbal information in their native language
Source: PLoS One. 2022 Oct 31;17(10):e0274901. doi: 10.1371/journal.pone.0274901 (PMC9621412; doi:10.1371/journal.pone.0274901)
Supplement: S1 Table — (PDF) [file pone.0274901.s001.pdf]

S1 Table. Scoring system used to assess the knowledge on the medicines prescribed.

Knowledge area	Level of knowledge	Points to be given
1. Name of the drug	1a) Knows the generic or brand name of the drug	2
	1b) Describes only the appearance correctly	1
	1c) Unable to describe the drug	0
2. Indication for the use of the drug	2a) Knows the correct indication	2
	2b) Not sure of the exact indication or knows a related indication	1
	2c) Does not know the indication at all	0
3. Dose of the drug	2a) Knows the dose in units (e.g., milligram/ grams/ unit)	2
	2b) Knows only the number of tablets/ capsules/ inhalations to be taken at one time	1
	2c) Not sure/ aware of the dose	0
4. Details of administration	2a) Knows when to take (e.g., morning, twice a day or 8 hourly) and special instructions on how to take (e.g., after meals, with lot of water, seated position)	2
	2b) Knows only when to take	1
	2c) Does not know details of administration	0
5. Aware of any additional facts about the medicine e.g. common side effects and what to do with those, or any special storage requirements etc.	5a) Knows at least one common side effect and what to do if it occurs (e.g., Hypoglycaemia with anti-diabetics and to take some sugar, take meals regularly etc.), store insulin in fridge.	2
	5b) Knows only a side effect but not what to do about it.	1
	5c) Unaware of any additional facts	0
